# Lacosamide induced Brugada I morphology in the setting of septicemia: A case report

**DOI:** 10.1097/MD.0000000000025577

**Published:** 2021-05-07

**Authors:** Robert Goodnough, Adina Badea, Curtis Geier, Kara L. Lynch, Kathy T. LeSaint

**Affiliations:** a Department of Emergency Medicine, Baylor College of Medicine, Houston, Texas; b Department of Laboratory Medicine; c Department of Pathology and Laboratory Medicine, Lifespan Academic Medical Center and Warren Alpert Medical School of Brown University, Providence, Rhode Island; d Department of Clinical Pharmacy; e Department of Emergency Medicine, University of California San Francisco, San Francisco, California.

**Keywords:** Brugada, lacosamide

## Abstract

**Introduction:**

Brugada syndrome may be unmasked by non-antiarrhythmic pharmaceuticals or drugs. Lacosamide is an antiepileptic agent with a novel mechanism of sodium channel inhibition and has the potential to cause cardiac sodium channel blockade.

**Patient concerns:**

In this report, we describe the case of patient with a history of a seizure disorder who presented with Brugada I electrocardiogram morphology in the setting of septicemia.

**Diagnosis:**

Brugada I electrocardiogram morphology was unmasked by lacosamide antiepileptic monotherapy.

**Interventions:**

Lacosamide therapy was discontinued.

**Outcomes:**

Normalization of the electrocardiogram and resolution of Brugada morphology occurred on hospital day 1.

**Conclusion:**

Caution should be exercised in the use of lacosamide in those at risk for conduction delay, or in combination therapy with medications that impair renal clearance, metabolism of lacosamide, or that display inherent sodium channel blocking properties.

## 1 Introduction

Drug-induced Brugada I morphology on electrocardiogram (ECG) may be used to diagnose Brugada syndrome by antiarrhythmic agents. The classic ST segment elevation in the Brugada syndrome may also be unmasked by non-antiarrhythmic pharmaceuticals such as tricyclic antidepressants or lithium, or by illicit drugs such as cocaine.^[1]^ Lacosamide is an antiepileptic agent with a novel mechanism of sodium channel inhibition compared with other antiepileptic or antiarrhythmic medications.^[2,3]^ In prior reports, lacosamide has potentially contributed to cardiac sodium channel blockade in combination with other medications, though its role as a sole agent is limited to massive overdose.^[2,4,5]^ Here, we present a case of Brugada I ECG morphology that was unmasked by lacosamide antiepileptic monotherapy in a patient with septicemia. Informed consent was obtained from the patient for the purpose of publication.

## 2 Case presentation

**Figure 1:**
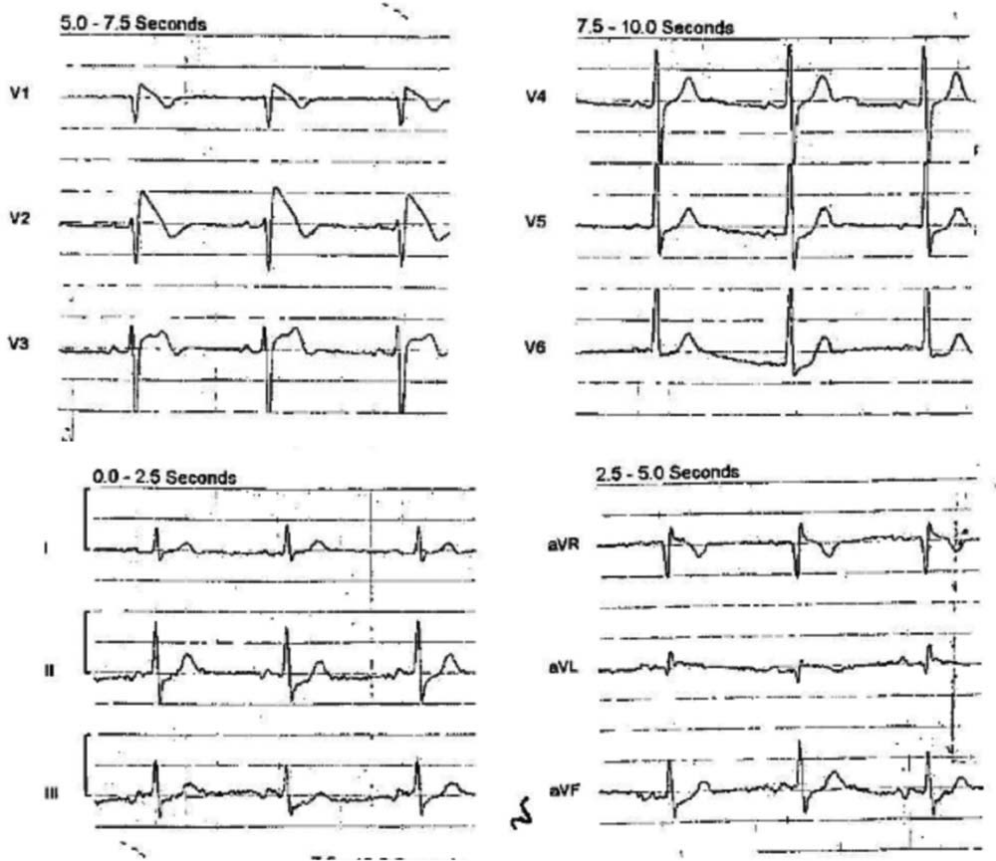
Pre-hospital electrocardiogram obtained on day of emergency department presentation.

An 83-year-old man with a history of chronic urinary retention due to benign prostatic hypertrophy and seizure disorder on daily lacosamide therapy developed fever and rigors in the late evening. Upon first responder arrival, the patient was alert, but tachycardic with a low systolic blood pressure (96 mmHg). A 12-lead pre-hospital ECG (Fig. 1) was interpreted as precordial ST elevation and the patient was transported to the nearest ST elevation myocardial infarction (STEMI) receiving center.

In the emergency department, the patient's vital signs were as follows: temperature 39.2 °C, heart rate 102 beats/min, blood pressure 109/65 mmHg, respiratory rate 18 breaths/min, and oxygen saturation 98% on 2 L of oxygen by nasal cannula. The patient denied any chest pain or shortness of breath. Physical examination was notable for a chronic indwelling Foley catheter. A chest radiograph displayed patchy bibasilar opacities. A complete blood count and comprehensive metabolic panel were remarkable only for mild anemia (hemoglobin 11.4 g/dL; normal 13.3–17.7 g/dL). Lactate was 3.6 mmol/L (normal <2 mmol/L), without acidosis or anion gap. The serum troponin was 0.06 ng/mL (normal <0.04 ng/mL), which peaked 16 hours after presentation at 0.29 ng/mL. A urinalysis showed pyuria and subsequent cultures of urine and 2 blood samples grew *Klebsiella oxytoca*.

At presentation, the Cardiology service was consulted regarding the possible STEMI on pre-hospital ECG and agreed that, given the patient's lack of symptoms consistent with acute coronary syndrome and the type I Brugada ECG pattern, the presentation was inconsistent with coronary occlusion. The ECG pattern was attributed to lacosamide effect and lacosamide was discontinued. The patient was transitioned to levetiracetam for seizure prophylaxis. He was admitted to the hospital for septicemia and treated with normal saline, levofloxacin, and ertapenem. Additionally, he was started on daily aspirin and atorvastatin.

Further seizure history was elicited. The patient had experienced 2 generalized seizures in his lifetime, with the first episode occurring 7 months prior for which he was initiated on phenytoin. In the subsequent month, he was transitioned from phenytoin to lacosamide 100 mg twice daily due to adverse effects of confusion and hallucinations. On the day of emergency department presentation, the patient had taken lacosamide for 6 months and his last dose was approximately 1 hour prior to arrival. Past documentation of ECGs prior to this event revealed that 6 months prior, after initiation of lacosamide therapy, a screening ECG showed sinus rhythm with J-point elevation in V2 and saddle back morphology, with normal PR, QRS, and QTc intervals.

Other than on arrival at emergency department, the patient had only one additional documented fever to 39.3 °C at 48 hours since presentation during this hospitalization. The patient's ECG pattern showed persistent ST elevation with a downsloping segment through 23 hours post-presentation, similar to pre-hospital findings. Normalization of the ECG and resolution of Brugada morphology was demonstrated at 54 hours after presentation. The patient was discharged on hospital day 5.

## 3 Discussion

Lacosamide is an antiepileptic agent used for partial onset seizures and works by enhancing slow activation of the SCN5A sodium channel.^[3]^ Less than 15% is protein bound in the plasma, it has a small volume of distribution (0.6 L/kg), and its elimination half-life is 13 to 16 hours. Forty percent of the drug excreted is unchanged in the urine.^[3]^

**Figure 2:**
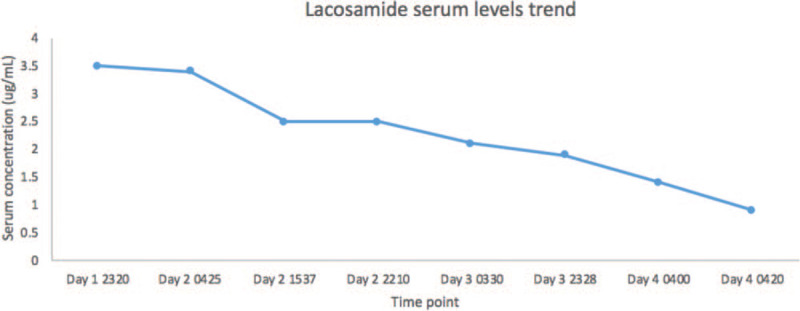
Serial lacosamide serum levels over time (therapeutic range: 2.5–18.0 μg/mL).

Lacosamide displays 10% to 20% inhibition of the SCN5A cardiac sodium channel at the upper limit of the therapeutic range, which increases in a dose dependent manner; mutations of SCN5A have been associated with Brugada syndrome and sudden death.^[2,6]^ Previous reports have suggested a role for therapeutic lacosamide induction of ventricular tachycardia during co-administration with other sodium channel blockers.^[2,4]^ A recent report demonstrated lacosamide-induced QRS prolongation and seizure after a likely massive overdose in a 48-year-old woman.^[5]^ In contrast to these reports, our patients only ingested one agent with sodium channel inhibiting properties, at therapeutic dosing, confirmed by liquid chromatography-high resolution mass spectrometry comprehensive testing and quantification (Fig. 2). Serial quantification confirmed clearance of lacosamide from the patient's serum that paralleled the resolution of Brugada morphology.

Our case illustrates the importance of recognition of Brugada I ECG morphology, which was mistaken for STEMI by first responders, and also shows the importance of recognition of medications and drugs that may invoke Brugada I pattern. This patient did not have a prior personal or family history of Brugada syndrome or syncope. The patient's history did not suggest overdose, and confirmatory testing demonstrated a serum drug concentration in the therapeutic range at the time of Brugada I ECG morphology. Recognizing the likely role of lacosamide, it was discontinued and the corresponding trend of ECGs over time demonstrated normalization.

A limitation of this report was the inability to perform genetic testing to exclude underlying mutations consistent with inherited sodium-channelopathy. ECGs prior to the patient's presentation had shown an incomplete right bundle branch block pattern, which may represent a normal variant or incomplete penetrance of a Brugada mutation which was unmasked by lacosamide-induced sodium channel blockade. Another limitation is that Brugada I morphology may be unmasked by fever. However, the pattern was not noted in prior presentations or ECGs, including during an admission for urosepsis in the preceding year, suggesting that the ECG morphology at presentation were likely, albeit not definitely, drug-induced. In addition, the morphology did not necessarily correlate with timing of the 2 documented fevers during the patient's hospital course further supporting lacosamide's role in producing the pattern.

This patient was treated with drug discontinuation and supportive measures, but it is warranted to consider that the pharmacokinetics of lacosamide may make it amenable to extracorporeal removal, especially in the setting of severe toxicity or impaired renal clearance; a prior study suggested 50% removal of lacosamide and its O-desmethyl metabolite in a 4-hour hemodialysis session.^[3,5]^

Recognition of lacosamide-induced Brugada I pattern had direct clinical relevance, as it allowed avoidance of unnecessary cardiac catheterization, prognostication for the patient, and avoidance of defibrillator placement, as drug-induced Brugada I is regarded as having a better prognosis than the spontaneous ECG morphology, though the former is not without risk.^[7]^ It is of particular relevance for providers to know to avoid lacosamide therapy in patients with Brugada syndrome.

## 4 Conclusions

This case highlights the risk for cardiac sodium channel blockade in lacosamide monotherapy, even in therapeutic dosing. Caution should be exercised in the use of lacosamide in those at risk for conduction delay, or in combination therapy with medications that impair renal clearance, metabolism of lacosamide, or that display inherent sodium channel blocking properties.

## Author contributions

**Conceptualization:** Robert Goodnough, Curtis Geier, Kathy T. LeSaint.

**Investigation:** Adina Badea, Kara L. Lynch.

**Methodology:** Robert Goodnough, Adina Badea, Curtis Geier, Kara L. Lynch, Kathy T. LeSaint.

**Supervision:** Kathy T. LeSaint.

**Writing – original draft:** Robert Goodnough, Kathy T. LeSaint.

**Writing – review & editing:** Robert Goodnough, Adina Badea, Curtis Geier, Kara L. Lynch, Kathy T. LeSaint.
